# The effect of virtual reality-based balance training on motor learning and postural control in healthy adults: a randomized preliminary study

**DOI:** 10.1186/s12938-018-0550-0

**Published:** 2018-09-18

**Authors:** Thunyanoot Prasertsakul, Panya Kaimuk, Wipawee Chinjenpradit, Weerawat Limroongreungrat, Warakorn Charoensuk

**Affiliations:** 1 Department of Biomedical Engineering, Faculty of Engineering, Mahidol University, Nakhon Pathom, 73170 Thailand; 2 Physical Medicine and Rehabilitation, Cardiac Rehabilitation, Bumrungrad International Hospital, Bangkok, Thailand; 3 College of Sports Science and Technology, Mahidol University, Nakhon Pathom, Thailand; 4 Department of Electrical Engineering, Faculty of Engineering, Mahidol University, Phuttamonthon 4 Road., Nakhon Pathom, 73170 Thailand

**Keywords:** Balance training, Virtual reality, Physical balance exercise, Motor learning, Balance performance

## Abstract

**Background:**

Adults with sedentary lifestyles seem to face a higher risk of falling in their later years. Several causes, such as impairment of strength, coordination, and cognitive function, influence worsening health conditions, including balancing ability. Many modalities can be applied to improve the balance function and prevent falling. Several studies have also recorded the effects of balance training in elderly adults for fall prevention. Accordingly, the aim of this study is to define the effect of virtual reality-based balance training on motor learning and postural control abilities in healthy adults.

**Methods:**

For this study, ten subjects were randomly allocated into either the conventional exercise (CON) or the virtual reality (VR) group. The CON group underwent physical balance training, while the VR group used the virtual reality system 4 weeks. In the VR group, the scores from three game modes were utilized to describe the effect of motor learning and define the learning curves that were derived with the power law function. Wilcoxon Signed Ranks Test was performed to analyze the postural control in five standing tasks, and data were collected with the help of a force plate.

**Results:**

The average score was used to describe the effect of motor learning by deriving the mathematical models for determining the learning curve. Additionally, the models were classified into two exponential functions that relied on the aim and requirement skills. A negative exponential function was observed in the game mode, which requires the cognitive-motor function. In contrast, a positive exponential function was found in the game with use of only the motor skill. Moreover, this curve and its model were also used to describe the effect of learning in the long term and the ratio of difficulty in each game. In the balance performance, there was a significant decrease in the center of pressure parameters in the VR group, while in the CON group, there was a significant increase in the parameters during some foot placements, especially in the medio-lateral direction.

**Conclusion:**

The proposed VR-based training relies on the effect of motor learning in long-term training though different kinds of task training. In postural analysis, both exercise programs are emphasized to improve the balance ability in healthy adults. However, the virtual reality system can promote better outcomes to improve postural control post exercising.

*Trial registration* Retrospectively registered on 25 April 2018. Trial number TCTR20180430005

**Electronic supplementary material:**

The online version of this article (10.1186/s12938-018-0550-0) contains supplementary material, which is available to authorized users.

## Background

The incidence of falls can occur in people of all ages and is not exclusively restricted to the elderly population [1]. Although the causes of falls are different for each age group, the decline in balance ability is a major factor for the high risk of falls. In older people, the decline in balance ability may occur due to physiological deterioration, pathological factors, problems of ambulation, and endurance reduction [2–7]. In addition, the physical activity level of children and middle-aged adults has decreased due to the development of technology, which has resulted in restriction of movement. This has led to the worsening of health conditions due to the deterioration of the neurotransmitter system [8] and muscle mass and strength [6, 9], giving rise to chronic diseases [10] as well as cognitive decline [11], which may induce a higher risk of falls in the future. People who suffer from these tend to get injured easily, which results in worsening of self-efficacy and functional dysfunction, even though they are disturbed by a small disturbance [12, 13]. Increasing physical activity, such as exercise, has a positive effect on several aspects, including postural stability and falling prevention [9].

Exercising is important, as it improves humans’ individual or systematic system, which is related to balance performance [2, 9, 13–17]. Exercises employ help prevent physiological deterioration by increasing strength and endurance of the body. For example, challenging the sensory system during postural tasks can enhance balance ability by reweighting the functional sensory inputs [18]. However, significant differences have been observed among various exercise programs, and some exercises have little effect on the balance function [9, 17–19]. Balance exercise programs may be made ineffective because of several reasons. First, various physiological systems are used to achieve the postural task [20, 21]. Second, the activities, which require balancing ability, can be achieved by coordinating between motor skills and cognitive activities [15]. Moreover, the training program with clinical guidelines is more effective than the program without any instruction [18]. Therefore, a combination of the exercise approach and the feedback during training process is used to improve the body’s functional ability, including balance performance [18, 22].

Using the gaming with the biofeedback system, such as the virtual reality (VR) system, is widely used for rehabilitation [12, 23–25]. It is due to the fact that the VR system can make the treatment more interesting, reduce the difficulty of rehabilitation, and increase safety [25–27]. One advantage of VR-based training is that this technology allows altering the neural organization, encouraging neuroplastic changes in neurological patients [26], reducing the fear of falling, and transferring into the real-world task through motor learning [28]. However, some VR-based balance training requires a specific balance platform, including Wii Fit balance board, to supply the sensory feedback information that may be restricted during the training process due to the requirement of a specific movement [18]. For this reason, popular sensors, e.g., the Microsoft Kinect sensor, have been used to show improvement in balance ability in several studies. This is due to the fact that Kinect sensor provides three-dimensional positions without using markers. These positions are used as input for the VR-system to improve balance function and reduce the fear of falling in older adults [29, 30].

In several studies, there were significant differences in clinical balance measures among participants who had trained with the help of conventional balance exercises, including the VR system [18, 27, 31, 32]. Additionally, most studies focused on their applications in improving balance for patients with neurological disorders [33–36] or elderly people [18, 25, 28]. Therefore, the aim of this study is to investigate the effects of VR-based balance training in healthy adults through motor learning and postural control. The questions included in the proposed study are (a) how does the VR-based balance exercise rely on the effect of motor learning? (b) how do the different exercise modalities influence the impairment of balance ability through comparison of balance performance before and after exercise? We hypothesize that the VR system affects postural control through motor learning. In addition, both balance exercise programs influence the postural control, but the balance performance in the VR-based balance exercise is better than the outcome of the conventional exercise.

## Methods

### Participants

The experiment in this study was designed as the pilot study. Community-dwelling healthy adults around the area of Mahidol University were recruited for the study. The inclusion criteria were (a) 40–60 years of age, (b) no history of injuries or diseases that influence balance function, (c) no intake of medications that affect postural control system, at least 12 h prior to the experiment, (d) no alcohol consumption 12 h prior to the experiment. The exclusion criteria were (a) individuals with dependent ambulation, (b) individuals who cannot communicate in the Thai language, and (c) individuals who have any disease that affects balance function.

Prior to data collection, all participants signed informed consent, which was approved by the Mahidol University Central Institutional Review Board (MU-IRB: 2014/112.1508). Demographic data and health information of the participants were obtained, following which they were randomly categorized into two groups, the virtual reality exercise (VR) group and the conventional balance exercise (CON) group, by blindly drawing a sealed piece of paper. The VR group (${n}= 5$) received the dual-task virtual-reality balance training system (DTVRBT), while the CON group (${n} = 5$) was assigned the conventional balance exercise.

### Protocol

The experimental protocol comprised three steps: the pre-test of balance performance, the balance training session, and the post-test for the evaluation of the balance ability after training. In the study, five standing tasks, including standing unsupported with eyes open (EO) and close (EC) conditions, standing with both feet together, tandem, and one-leg stance were evaluated. Results of balance evaluation in each task were collected for 10 s/trial, with three trials, and the testing focused on the dominant leg in tandem and the one-leg stance. The total of time duration for data analysis was 30 s. In this study, the MatScan^®^ model 3150 (Massachusetts, USA) was used to assess the center-of-pressure (CoP) in the anterior–posterior (AP) and medio-lateral (ML) directions with the sampling rate was 64 Hz. The data of each subject was exported with the Sway Analysis Module (SAM™). The training session started after 1 week of completion of the pre-test, and the post-test was performed within 1 week of finishing the training session. All participants received twelve 45-min sessions of training in the DTVRBT or the conventional balance exercise program. Moreover, three sessions were held per week for a period of 4 weeks. The same physical therapist conducted the training for both groups.

### Dual-task virtual reality balance training system

**Fig. 1:**
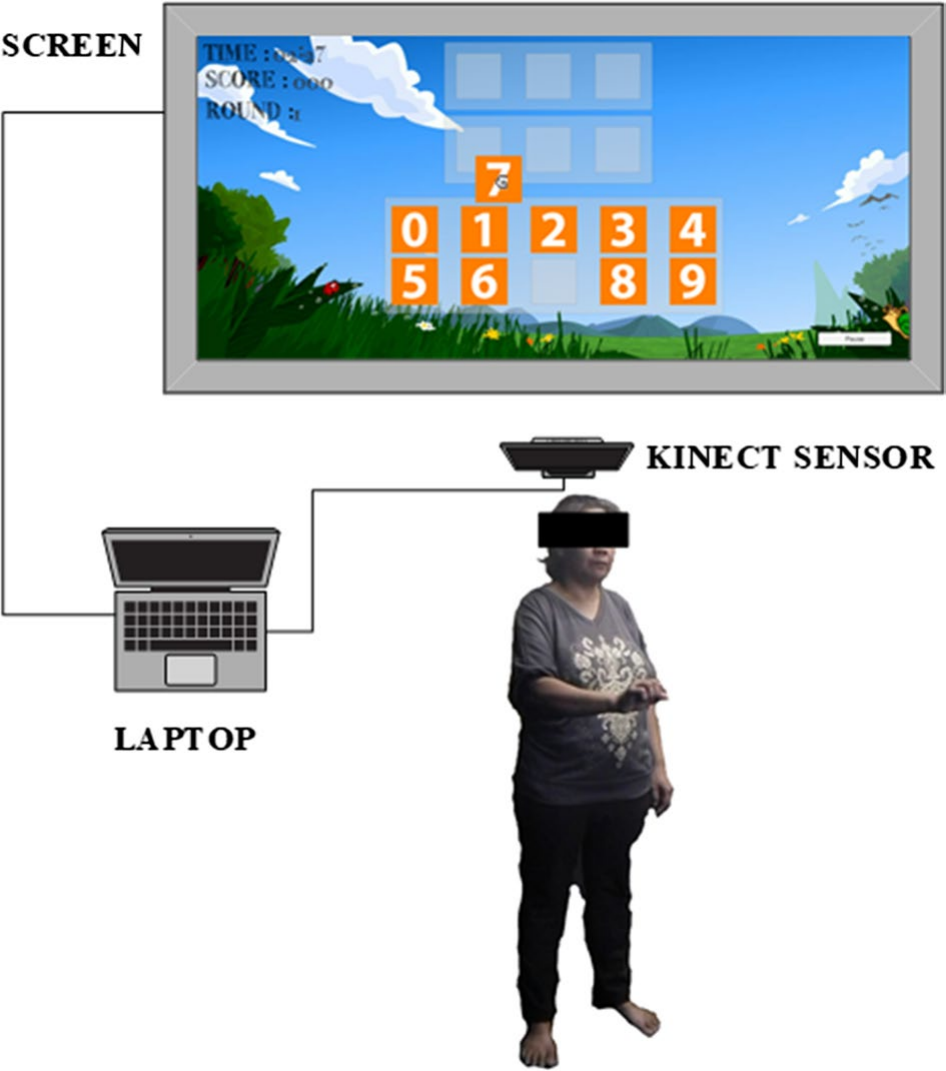
The process of interaction in the virtual environment by the Kinect sensor

The DTVRBT consists of a laptop and the Kinect sensor (Washington, USA) as shown in Fig. 1. This sensor can construct 3D images from the functional integration of two components, an RGB camera and an infrared sensor [29, 32]. The 3D information from this sensor allows users to interact with the object in the virtual environment. In this study, the virtual environment was created with the Unity3D^®^ version 5.3.2. (San Francisco, USA).

**Fig. 2:**
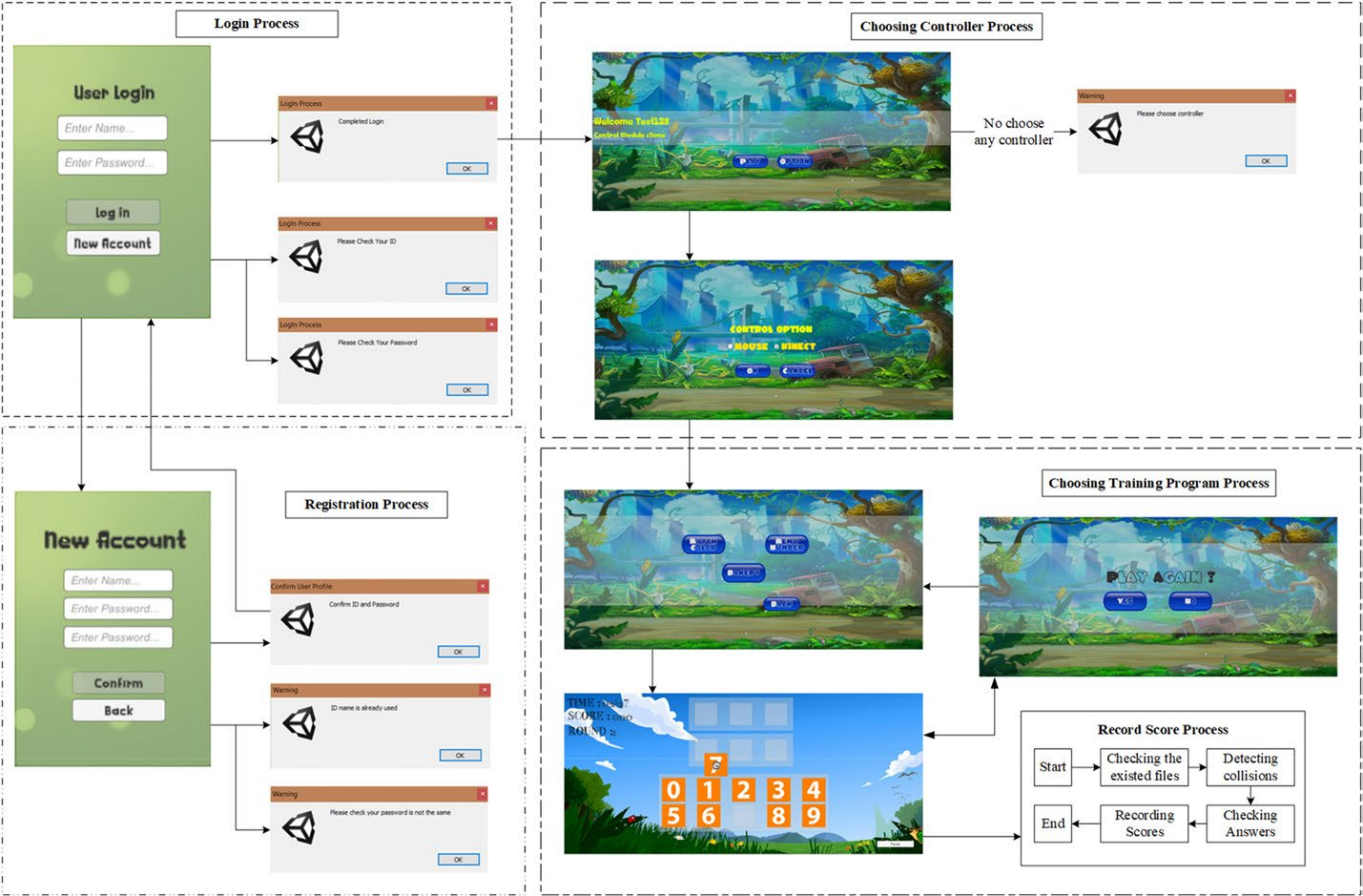
The process of collecting the score for the dual-task virtual reality balance training (DTVRBT) system

**Fig. 3:**
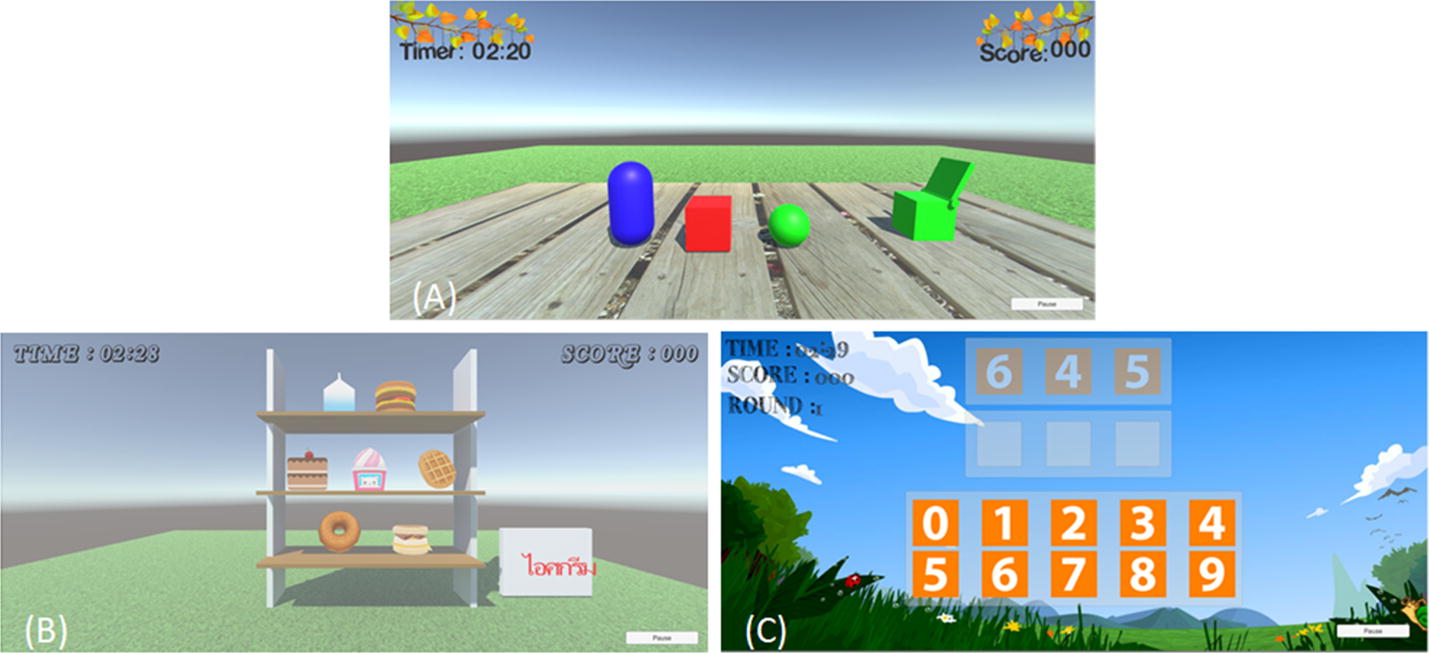
Interface of three game modes consisting of **A** matching color, **B** bakery, and **C** memo number

In this study, the scores of each individual were used to describe the performance of training with the virtual reality system. The process of recording the score consisted of four main steps, as shown in Fig. 2. First, all users were required to log in before proceeding with the other steps. In this process, a user profile would be created as a folder that contained the files of training for each day. This process required usernames and passwords. If an incorrect username or password was entered, the system would request a new username or password. Unique users needed to create their usernames and passwords before beginning the login process. The program would warn users when they entered the same name as one that had been previously registered, and two passwords could never be the same. After finishing the login process, the users had to choose the controller and game mode of training. In the game mode, the DTVRBT consisted of three modes of training, such as matching color, bakery, and memo number (refer to Fig. 3). The descriptions of each game mode are presented in Table 1.

**Table 1:** The descriptions of the tasks in the dual-task virtual reality balance training (DTVTBT) system

Names of game mode	Type of task	Weight shifting	Task description
Matching color	Single-task	ML	Waiting for randomized the box’s color. The player moves hand to control the virtual hand inside the virtual environment. They have to place and grasp the hand on the object that has the same box’s color and move the hand to the box
Bakery	Single-task	AP and ML	Waiting for randomized name and position of order. The player moves their hand to control the virtual hand inside the virtual environment. They have to place and grasp the hand on the object that has the same item’s name and move the hand to the order
Memo number	Dual-task	AP and ML	Waiting for randomized three numbers, after which these numbers fade. The player moves their hand to control the virtual hand inside the virtual environment. They place and grasp the hand on the number, move the hand to the slot by sequencing order, and release the hand to insert the number into the slot

In Table 1, the game modes are shown to be separated into two types of training tasks, i.e., single or dual task. In the single-task training, rapid arm movement is required to complete the task, unlike the dual-task training. It is due to the fact that the dual-task training in this system requires the cognitive-motor function to achieve the goal. In order to complete the training, the players would have to stand and use the dominant arm while performing the arm movement task. The matching color is the first program, which is the easiest mode of training. This game mode requires arm movements in the horizontal direction to get an object and place it at the target position. The bakery is the second program to practice balance training, and the task is more complex than the first program. This game mode requires arm movements in the vertical and horizontal directions to complete the task. The player must place the object at the assigned area if the object’s name appears. In this game, the positions of objects on the shelf are randomized in each round to avoid learning and remembering. However, the difficulty level of this game mode is lesser than that of the memo number, which is the last program in the virtual reality-based training session. This game mode also requires arm movements in both directions, same as the second game mode. The cognitive task, i.e., recalling of a sequence of three numbers, is added during the performance of the postural task. The process of obtaining the score of these game modes is that if there is a correct answer, the score increases by 10 points, except for the memo number. The score of each number in the memo number accounts for 10 points. Therefore, the total score in each round is 30 points. The scores in each round from each game mode are recorded in the form of a text file. For balance training with this protocol, six trials for each game were conducted, and each trial required around 2 min 30 s of time. The total duration for training was 45 min, and the participant could rest between games for 1 min.

### Conventional balance exercise

CON received the physical balance exercise program that consisted of two types of exercise: static and dynamic balance exercises. In this study, the proposed program was modified from Silsupadol et al. [2]. The static balance training consisted of the following: (a) standing with feet apart with eyes open, followed by putting the feet together, (b) standing with feet apart with eyes closed, (c) standing with the dominant foot in front of the other foot (or tandem stance), (d) disturbance during quiet standing, and (e) standing on firm surface (e.g. wooden or cement floor) or unstable surface (e.g. foam, grass, or sand). The protocol in dynamic balance training condition consisted of (a) double leg stance on firm surface while holding a glass of water, (b) tandem stance while turning the hand quickly, (c) double leg stance while reaching in any direction, and (d) throwing and getting a ball while in the standing position. Two dynamic tasks, i.e. the reaching task and the ball throwing task, boosted the balance function in AP and ML directions. Each participant was instructed by a physical therapist to perform the exercises at home, and they had to maintain records in a diary of the exercises that were performed in each session.

### Outcome measures

The game performance in this study could be presented in the form of the score that was obtained when a participant completed the training program in each session. The player needed to respond by placing objects in the assigned position. Accordingly, the efforts of the player could be reinforced through the immediate feedback information during the game itself. In order to determine game performance, the average of scores from the six trials of all the participants in each game mode were utilized to describe the effect of motor learning with the learning curve that was derived from the power law function [37]. Generally, the power law function was a quantitative form of the learning curve which predicted the time or speed to the practice trials [38]. It was due to the fact that this function was widely applied in speed task [39]. The increased speed movement indicated the occurrence of getting more score. To determine each model, a simple formulation, as shown below, was considered. Where *y*(*n*) was estimated values to fit a learning curve, *n* was the session number, and *a*, *b* and *c* were constants which were determined by fitting score. Three mathematical models were determined using the MATLAB software (Massachusetts, USA).

$$\begin{aligned} y(n)=a(n^{b})+c \end{aligned}$$

In postural analysis, the trajectory of CoP on the AP and ML directions was used to investigate balance performance. All CoP parameters were also obtained using MATLAB software. The CoP parameters including the total excursion (TOTEX) on AP, ML, and statokinesigram (SK) directions, CoP range on AP and ML directions, and 95% confidence ellipse area (Area$_{E}$) were considered to evaluate the balance ability. First, TOTEX was defined as the total distance of CoP traveling that was referred to sway path in each direction. Second, CoP range represented the maximum amplitude of CoP on AP and ML direction. The maximum and minimum amplitude values in each direction could provide the CoP range. Last, the Area$_{E}$ illustrated the 95% of total area contained in both directions which were fitted by an ellipse [40, 41]. A greater CoP range indicated the worse stability [41] which resulted in higher TOTEX. Thus, the higher TOTEX encouraged on lower precision of CoP movement [42], larger Area$_{E}$, and poorer balance performance [41]. The calculation of these parameters has been described in Prieto et al. [43], except for the Area$_{E}$. This area was defined by Brog [44]. Before calculating these parameters, the trajectory of CoP in any task was normalized with mean subtraction and filtered with the second order of Butterworth and 4 Hz. of the cut-off frequency. After acquiring the parameters, the average of the three trials from the participants in the CON or VR groups was defined.

### Statistical analysis

In CoP analysis, data of this report was described in the form of group mean and standard deviation (SD). The statistical analysis was performed in PASW Statistics 18 (Illinois, USA). The normality of data was tested by Shapiro–Wilk test. The Wilcoxon Signed Ranks Test was employed to compare the effects of both modalities in each group. The significance level at p-value $(p) < 0.05$ was accepted and reported in the study.

## Results

One participant from each group was excluded, as they could not complete the training protocol. Therefore, there were four participants in the VR group (age = $51.5 \pm 6.61$ years, height = $159.0 \pm 6.83$ cm, weight = $68.5 \pm 10.47$ kg) and the CON group (age = $55.0 \pm 5.72$ years, height = $155.25 \pm 5.89$ cm, weight = $56.45 \pm 1.45$ kg).

### The mathematical models of motor learning

**Fig. 4:**
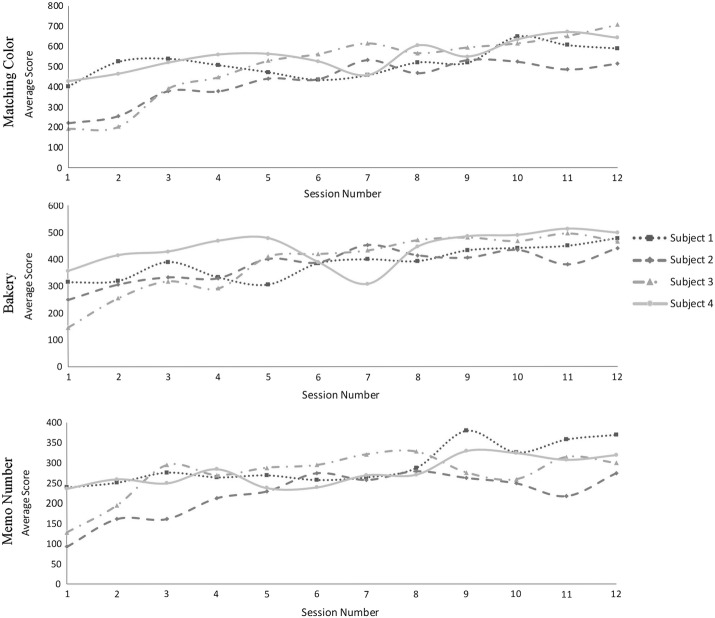
The average scores from three games, i.e., matching color, bakery, and memo number, in twelve training sessions for four participants

Our findings showed that the average scores from all participants were gradually increasing for all games. In addition, the matching color game mode had the highest average score, whereas the memo number game mode had the lowest average score at the last session of training for all participants, as shown in Fig. 4. Although this value would indicate the effectiveness of learning and training performance owing to training with the virtual reality system, a learning curve was used rather than using the average scores. Typically, the rate of learning curve depends on the range of exponents that are described in form of several functions, such as the hyperbolic and exponential functions. However, the power law is acceptable, as this function can fit the learning curve better than the other functions [37]. Accordingly, this function was utilized to fit the learning curves of the three game modes, as described below.

$$\begin{aligned} y(n)= & {} 510.9 (n^{0.185})-198.5\\ y(n)= & {} 311.6 (n^{0.1973})-38.53\\ y(n)= & {} -770.9 (n^{-0.07637})+947.4 \end{aligned}$$

**Fig. 5:**
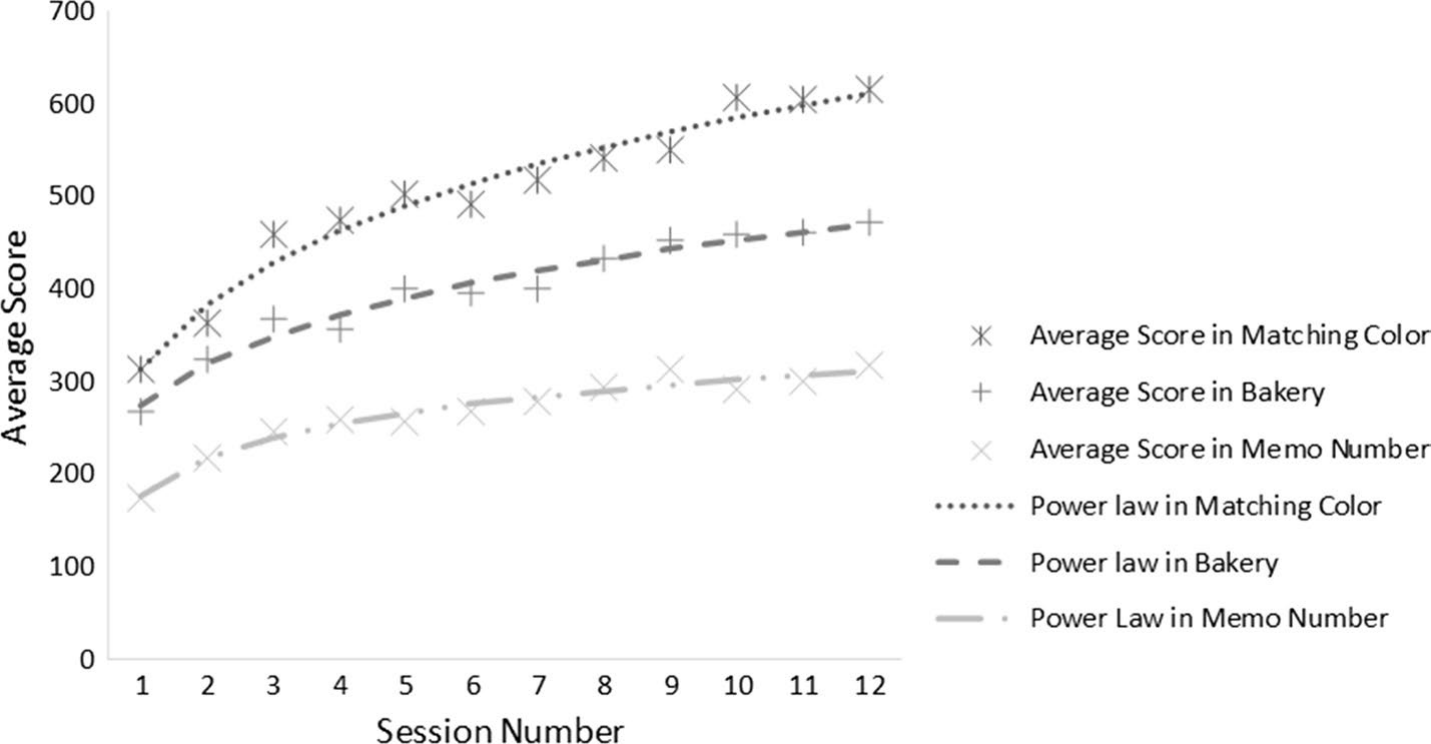
The average score and fitted curves from three equations of the three game modes

The first, second, and third equations present the mathematical models to estimate the curve for three game modes, the matching color, bakery, and memo number respectively. Three learning curves and their average scores are shown in Fig. 5.

The findings listed in Fig. 5 revealed that the scores from the matching color game mode had the highest scores, whereas the memo number game had the lowest scores for all sessions. In addition, the learning curve in the early sessions of training had a higher slope than at the end of training session for all game modes. Furthermore, all curves showed continuous learning in all game modes, although these curves have tended to be the plateau phase (refer to Additional file 1).

### The outcomes of balance performance

Our results found that most of the CoP parameters in the VR group decreased after training, except in the EO case. In addition, there was no significant difference (ns) in the CoP parameters for the ML direction, except the EC and tandem stance, as shown in Table 2.

**Table 2:** Mean and standard deviation (SD) of CoP parameters between pre-test and post-test in the VR group

Standing task	CoP parameter	Direction	Pre-test mean (SD)	Post-test mean (SD)	Significance
EO	Area$_{E}$ (mm$^2$)	SK	328.01 (105.09)	222.52 (173.97)	ns
		SK	82.81 (9.44)	84.77 (13.53)	ns
	TOTEX (mm)	AP	63.46 (11.92)	64.81 (13.31)	${p < 0.05}$
		ML	39.81 (5.43)	42.08 (6.89)	ns
	RANGE (mm)	AP	14.52 (4.72)	16.96 (6.16)	${p < 0.05}$
		ML	8.00 (4.27)	6.23 (3.70)	ns
EC	Area$_{E}$ (mm$^2$)	SK	313.22 (309.38)	90.34 (64.55)	${p < 0.05}$
		SK	136.80 (58.30)	105.42 (6.39)	${p < 0.05}$
	TOTEX (mm)	AP	114.97 (54.14)	84.08 (7.93)	${p < 0.05}$
		ML	54.15 (19.80)	47.76 (6.83)	${p < 0.05}$
	RANGE (mm)	AP	28.04 (13.78)	15.03 (3.20)	${p < 0.05}$
		ML	9.05 (5.84)	5.62 (2.31)	${p < 0.05}$
Feet together	Area$_{E}$ (mm$^2$)	SK	1233.67 (719.06)	845.41 (518.07)	${p < 0.05}$
		SK	187.92 (76.19)	180.43 (45.69)	${p < 0.05}$
	TOTEX (mm)	AP	107.97 (56.37)	93.06 (14.32)	${p < 0.05}$
		ML	130.31 (41.47)	134.96 (44.64)	ns
	RANGE (mm)	AP	23.25 (8.28)	20.00 (6.37)	${p < 0.05}$
		ML	30.42 (9.88)	30.01 (11.64)	ns
Tandem	Area$_{E}$ (mm$^2$)	SK	1251.15 (1175.04)	763.13 (763.13)	${p < 0.05}$
		SK	292.49 (101.26)	286.03 (71.88)	${p < 0.05}$
	TOTEX (mm)	AP	189.12 (60.49)	179.99 (52.94)	${p < 0.05}$
		ML	183.00 (88.22)	180.67 (70.56)	${p < 0.05}$
	RANGE (mm)	AP	38.29 (15.60)	26.89 (13.22)	${p < 0.05}$
		ML	29.98 (12.89)	29.88 (16.73)	${p < 0.05}$
One leg	Area$_{E}$ (mm$^2$)	SK	1554.52 (677.93)	1043.91 (249.42)	${p < 0.05}$
		SK	381.30 (110.24)	379.47 (56.50)	${p < 0.05}$
	TOTEX (mm)	AP	252.20 (91.88)	231.72 (38.10)	${p < 0.05}$
		ML	239.07 (50.79)	255.56 (45.84)	ns
	RANGE (mm)	AP	42.54 (12.59)	33.41 (3.88)	${p < 0.05}$
		ML	37.76 (7.59)	34.29 (5.84)	ns

The results in the CON group differed from the VR group that had significant difference in some CoP parameters, as shown in Table 3. Although, the results showed that the CoP range, TOTEX in the ML direction, and the Area$_{E}$ significantly increased in most standing tasks, some other parameters significantly decreased after exercising. Furthermore, there was no significant difference for the one-leg stance.

**Table 3:** Mean and standard deviation (SD) of CoP parameters between pre-test and post-test in the CON group

Standing task	CoP parameter	Direction	Pre-test mean (SD)	Post-test mean (SD)	Significance
EO	Area$_{E}$ (mm$^2$)	SK	149.79 (53.54)	282.10 (208.18)	${p < 0.05}$
		SK	73.47 (7.78)	76.99 (7.88)	${p < 0.05}$
	TOTEX (mm)	AP	55.25 (10.28)	57.29 (11.3)	ns
		ML	36.97 (10.01)	40.52 (6.43)	${p < 0.05}$
	RANGE (mm)	AP	13.19 (3.23)	12.70 (3.71)	ns
		ML	5.00 (1.32)	8.95 (1.37)	${p < 0.05}$
EC	Area$_{E}$ (mm$^2$)	SK	127.27 (69.07)	128.04 (56.91)	ns
		SK	109.97 (6.07)	95.90 (16.59)	ns
	TOTEX (mm)	AP	87.27 (6.48)	74.49 (12.83)	${p < 0.05}$
		ML	49.96 (5.07)	45.62 (10.99)	ns
	RANGE (mm)	AP	16.35 (1.27)	14.80 (4.07)	${p < 0.05}$
		ML	7.87 (3.28)	8.31 (2.52)	ns
Feet together	Area$_{E}$ (mm$^2$)	SK	1330.04 (753.35)	1016.24 (62.89)	ns
		SK	167.95 (59.90)	181.52 (30.80)	ns
	TOTEX (mm)	AP	95.18 (36.42)	93.34 (29.30)	${p < 0.05}$
		ML	114.54 (42.79)	136.72 (16.96)	${p < 0.05}$
	RANGE (mm)	AP	23.22 (5.90)	19.62 (6.86)	${p < 0.05}$
		ML	26.40 (10.01)	30.28 (7.56)	${p < 0.05}$
Tandem	Area$_{E}$ (mm$^2$)	SK	709.80 (336.62)	910.40 (618.66)	ns
		SK	270.36 (80.78)	282.86 (99.83)	ns
	TOTEX (mm)	AP	202.89 (77.61)	176.21 (59.19)	${p < 0.05}$
		ML	134.15 (40.49)	183.26 (73.40)	${p < 0.05}$
	RANGE (mm)	AP	31.51 (15.05)	28.32 (5.99)	${p < 0.05}$
		ML	22.98 (3.48)	30.86 (13.11)	${p < 0.05}$
One leg	Area$_{E}$ (mm$^2$)	SK	1129.34 (576.60)	1107.59 (214.51)	ns
		SK	286.44 (58.69)	310.52 (45.74)	ns
	TOTEX (mm)	AP	193.57 (59.28)	194.27 (42.49)	ns
		ML	176.43 (20.57)	205.95 (23.20)	ns
	RANGE (mm)	AP	35.21 (14.88)	31.36 (7.99)	ns
		ML	29.42 (3.76)	31.46 (2.41)	ns

## Discussion

The proposed preliminary study investigates the effect of virtual reality-based training through two aspects: the effect on motor learning and postural control. The effect of motor learning depends on the required skill, which relates to the difference in game design. In this study, the game design was separated into single- and dual-task training. To understand the effect of different task training sessions, the average scores and their learning curves were used to describe the performance of motor learning for each game mode in case of all the participants who practiced with the virtual reality system. The exponent component in each mathematical model illustrated the different requirement skills during training with any mode. In addition, the learning curve and model were also used to determine the continual of motor learning and ratio of difficulty level. Furthermore, this preliminary study also investigated the effect of two modalities on healthy adults for short-term training (4 weeks). Our findings revealed that the virtual reality-based balance training affected balance improvement in several foot placements, while the enhancement of postural control for the CON group was observed in some standing tasks.

In the conventional balance exercise, the proposed program was designed to increase the challenge of balance ability through two approaches. First, reducing the base-of-support (BoS) or sensory input is one of four ways to increase the effectiveness of balance exercises. Second, the dual-task exercise, such as the hand–eye coordination with the balance task, also plays a role in challenging the postural control while performing a different task [45]. Although several studies have highlighted significant differences among subjects who practice with the conventional balance training program [2, 46–48], this program differs from training with virtual reality technology, as physical exercise does not provide the real-time feedback information [25, 26, 34, 49].

There are several reasons for which the virtual reality-based exercise program can improve various physiological functions. First, this program encourages the enhancement of motor learning. The improvement of this learning influences the reception of score, which is one of several approaches to represent the performance of this training. Many factors, such as the familiarity in the virtual environment, anticipation of required movements, and the improvement of sensorimotor, coordination and balance, add to the increasing score while practicing with this program for a long time [31]. However, the average scores could not illustrate the quality of learning. These values were used to determine the learning curve for describing the effect of training for the motor learning condition. Interestingly, our findings revealed that the exponent compartment in each model reflected the different requirement skills through different exponential functions. These functions can be categorized into two groups: positive and negative exponential functions. In this study, positive exponential function was obtained in two modes: the matching color and bakery. Both game modes were arranged in single-task training sessions, which required the arm movement function during standing. The negative exponential function was found in the memo number game mode that was added to the cognitive-motor training in the exercise program. Moreover, the learning curve and its model were also used to describe in more detail the effectiveness of game performance in long-term training for the motor learning and the ratio of difficulty level of these games.

The mathematical models that were derived from the power law function indicated the effect of continuous learning by observing the occurrence of plateau phase in the learning curve. Although the gradient slope in each game mode was decreasing in every session, our findings showed the tendency of plateau phase. In fact, the gradient of slope from three game modes was larger than zero (refer to Additional file 1). According to these findings, the learning curves from these game modes did not reach the mastery level, which showed the effect of continuous motor learning [31]. Continuous motor learning reflects the occurrence of lifelong activities that relate to experience-related brain plasticity due to the fact that the learner needs to repeat the physical practice of a given functional skill [11].

The relative difficulty of three game modes was due to other factors that could describe the game performance in different types of task training. This ration was computed by the exponents from two models. The ratio of difficulty between the matching color and bakery game modes was $0.185/0.1973 = 0.938$. In other game modes, the ratio of difficulty between matching color and memo number as well as between bakery and memo number were $0.185/0.076 = 2.422$ and $0.1973/0.076 = 2.583$, respectively. Based on these results, it is evident that the higher ratio of difficulty, the more difficult it is to gain the score. Our findings showed that the ratio between single-task game modes was the lowest ratio of difficulty. Accordingly, there were several causes that influenced more difficult level in the bakery game mode. First, the bakery game mode requires more directions of arm movements than the matching color game mode. Second, the bakery mode needs more directions of weight shifting that correspond to the direction of arm movements. Last, there are four assigned positions that are randomized to obtain the correct answer, in contrast to the matching color game mode that has only one target. It is due to the fact that the training with choice reaction time condition affects the increase of motor progress, which corresponds with the results of Kubichi et al. [50].

Interestingly, adding the cognitive-motor training into the exercise program causes a higher ratio of difficulty when compared to single-task training. There are two reasons for which the difficulty of postural task increases while the cognitive task is processing. First, the cognitive process, including the attention, concentration, working memory, and information processing speed, are the main aspects that rely on motor learning [11]. Second, the postural control function and other cognitive processing must share the cognitive resource. The performance in postural control can be impaired by a secondary cognitive task [51, 52]. For this reason, the challenge of balance training with the cognitive task can improve the performance of postural control, as shown in this study.

In the postural analysis, there are two balance exercise approaches to observe the effectiveness of different exercise programs. Our findings revealed that both balance exercise programs could be employed to improve the postural control under the CoP measurement of the five standing tasks. In the CON group, the results suggested that standing with eyes open had a significant difference in the Area$_{E}$. The increase of the CoP range on the ML direction resulted in the increase of TOTEX for all directions. In addition, we found that the significant difference of the CoP range on the ML direction was the only factor for increasingly significance in the Area$_{E}$. However, there was no significant difference in the same CoP and area parameters on the other standing tasks. Based on this result, the proposed conventional balance exercise could encourage the enhancement of postural control in only the standing task—the EO case. It was because this standing task was used in many exercises for this program. Moreover, the CoP range on the ML direction in the CON group was increased in all the standing tasks. We hypothesized that some activities that promoted the weight shifting in the ML direction, such as tandem stance and throwing and getting a ball, were applied in the program. For this reason, our findings were contradictory to those of other studies [53]. In addition, the proposed conventional exercise could not transfer the experience of training to other standing tasks due to the existence of no significant difference in the four standing tasks (see Table 3).

In the VR group, the improvement in postural control had been observed in all the standing tasks that differed from the CON group. There were decreased significant differences in all CoP parameters and Area$_{E}$ between pre-test and post-test balance evaluation, except in the EO case. The reduction of CoP parameters in most standing tasks indicated that the people in this group had better postural control [41, 53]. For this reason, it becomes evident that training with the proposed virtual reality system contributed to transferring the experience from the virtual world to the real-world situation. Interestingly, the findings suggested that the dual task virtual reality-based balance training allowed the participants to stand with feet apart, in contrast to the conventional balance exercise that provided several positions and activities to improve the balance function. Strong significant differences were obtained, especially in the standing tasks with low base-of-support (BoS). The improvement of postural control in the VR group corresponded with the findings of many studies [53, 54]. However, the area parameter in the EO case had no significant difference, as this pose was used while exercising with the virtual reality system, although the CoP range in the AP direction was increased and the range in the ML direction was decreased.

Several factors encourage the improvement of postural control due to the use of virtual reality-based balance training. First, the postural control in static condition requires the visual system to provide the feedback information to complete the balance task. It is because this information relates to position and orientation of the body’s position [55]. Second, exergame modality, which integrates with the virtual reality technology, requires the body movements during a game. The combination of both components allows users to assess augmented feedback in real-time while they perform specific tasks, which results in balance improvement. Third, virtual reality-based training can enhance the motor learning through the repeated accurate performance [32]. Fourth, specific movements, such as rapid arm movement, are used to maintain and improve balance ability [31, 50]. It is because using unilateral or bilateral arm movements can encourage the reactive responses in postural leg muscles. In addition, hand functions, such as reaching and grasping, are also necessary to recover the balance function and prevent falls [31]. Accordingly, the game design of this study corresponded to these articles by using the benefits of this rapid movement in order to promote better balance function. Based on these findings, the virtual reality system can encourage learning in two categories: through the motor learning and through balance improvement. Furthermore, our results revealed that both exercise programs effected changes in postural control, but the virtual reality system could provide better performance than the conventional exercise due to the reduction of CoP parameter after exercise training. For this reason, feedback information appears to be a main key for functional training, including the balance exercise training. However, there were some limitations in the proposed study due to the small sample size. Another limitation was the computational score in the virtual reality-based training, which focused on only one aspect, the correct direction of movement or answer. The score of balance ability was not included to calculate the score. Thus, increasing the sample size and adding the score of balance performance in the virtual reality-based balance training is recommended for future studies.

## Conclusion

The proposed study focuses on the effect of the virtual reality-based training on two conditions: the effects of motor learning and postural control. In motor learning, the mathematical models that are represented with learning curves are utilized to describe the effectiveness of the proposed exercise program with the virtual reality system. The findings reveal that the learning curves can be separated into two groups that relate to the aim in each game mode and requirement skills. In addition, the continue learning of motor skill also observe in these learning curves. In the postural analysis, the findings indicate that the balance ability from both groups can improve by both exercise modalities. However, the virtual reality-based exercise program can improve the postural control better than the conventional exercise that includes several standing tasks. This is evidenced by the fact that the CoP parameters in the virtual reality-based training are smaller than those of the conventional exercise. Based on the results of this study, it is evident that the virtual reality system can facilitate better postural control than the conventional exercise and contribute to falling prevention of healthy adults in the future.

## Additional file

**Additional file 1.** The gradient of slope determined from all sessions in each game. This excel file describes the way to compute the gradient of slope to define the plateau phase in each game. The scores from a fitted curve, using the power law function, are used to determine the slope.

## References

[1] Talbot LA, Musiol RJ, Witham EK, Metter EJ (2005). Falls in young, middle-aged and older community dwelling adults: perceived cause, environmental factors and injury. BMC Public Health.

[2] Silsupadol P, Siu KC, Shumway-Cook A, Woollacott MH (2006). Training of balance under single- and dual-task conditions in older adults with balance impairment. Phys Ther.

[3] Toraman A, Yildirim NU (2010). The falling risk and physical fitness in older people. Arch Gerontol Geriatr.

[4] Ferreira ML, Sherrington C, Smith K, Carswell P, Bell R, Bell M, Nascimento DP, Maximo P, Pereira LS, Vardon P (2012). Physical activity improves strength, balance and endurance in adults aged 40–65 years: a systematic review. J Physiother.

[5] Ungar A, Rafanelli M, Iacomelli I, Brunetti MA, Ceccofiglio A, Tesi F, Marchionni N (2013). Fall prevention in the elderly. Clin Cases Miner Bone Metab.

[6] Keller K, Engelhardt M (2013). Strength and muscle mass loss with aging process. Muscles ligaments Tendons J.

[7] Santilli V, Bernetti A, Mangone M, Paoloni M (2014). Clinical definition of sarcopenia. Clin Cases Miner Bone Metab.

[8] Seidler RD, Bernard JA, Burutolu TB, Fling BW, Gordon MT, Gwin JT, Kwak Y, Lipps DB (2010). Motor control and aging: links to age-related brain structural, functional, and biochemical effects. Neurosci Biobehav Rev.

[9] Skelton DA (2001). Effects of physical activity on postural stability. Age Ageing.

[10] Booth FW, Roberts CK, Laye MJ (2012). Lack of exercise is a major cause of chronic diseases. Compre Physiol.

[11] Cai L, Chan JSY, Yan JH, Peng K (2014). Brain plasticity and motor practice in cognitive aging. Front Aging Neurosci.

[12] Betker AL, Szturm T, Moussavi ZK, Nett C (2006). Video game-based exercises for balance rehabilitation: a single-subject design. ch Phys Med Rehabil.

[13] Cadore EL, Lodríguez-Mañas RR, Sinclair A, Izquierdo M (2013). Effects of different exercise interventions on risk of falls, gait ability, and balance in physically frail older adults: a systematic review. Rejuven Res.

[14] Tsang WW, Hui-Chan CW (2005). Comparison of muscle torque, balance, and confidence in older Tai Chi and healthy adults. Med Sci Sports Exerc.

[15] Shubert TE, McCulloch K, Hartman M, Giuliani CA (2010). The effect of an exercise-based balance intervention on physical and cognitive performance for older adults: a pilot study. J Geriatr Phys Ther.

[16] Newell D, Shead V, Sloane L (2012). Changes in gait and balance parameters in elderly subjects attending an 8-week supervised Pilates programme. J Bodyw Mov Ther.

[17] Lesinski M, Hortobagyi T, Muehlbauer T, Gollhofer A, Granacher U (2015). Effects of balance training on balance performance in healthy older adults: a systematic review and meta-analysis. Sports Med.

[18] Bao T, Carender WJ, Kinnaird C, Barone VJ, Peethambaran G, Whitney SL, Kabeto M, Seidler RD, Sienko KH (2018). Effects of long-term balance training with vibrotactile sensory augmentation among community-dwelling healthy older adults: a randomized preliminary study. J NeuroEng Rehab.

[19] Bisson E, Contant B, Sveistrup H, Lajoie Y (2007). Functional balance and dual-task reaction times in older adults are improved by virtual reality and biofeedback training. Cyberpsychol Behav.

[20] Massion J (1994). Postural control system. Curr Opin Neurobiol.

[21] de Oliveira CB, de Medeiros IRT, Frota NAF, Greters ME, Conforto AB (2008). Balance control in hemiparetic stroke patients: main tools for evaluation. J Rehab Res Dev.

[22] Sturnieks DL, George RS, Lord SR (2008). Balance disorders in the elderly. Clin Neurophysiol.

[23] Gil-Gomez JA, Llorens R, Alcaniz M, Colomer C (2011). Effectiveness of a Wii balance board-based system (eBaViR) for balance rehabilitation: a pilot randomized clinical trial in patients with acquired brain injury. J Neuroeng Rehabil.

[24] Kennedy MW, Schmiedeler JP, Crowell CR, Villano M, Striegel AD, Kuitse J. Enhanced feedback in balance rehabilitation using the Nintendo Wii Balance Board. In: 2011 IEEE 13th international conference on e-health networking, applications and services. 2011. p. 162–168. 10.1109/HEALTH.2011.6026735

[25] Lange BS, Requejo P, Flynn SM, Rizzo AA, Valero-Cuevas FJ, Baker L, Winstein C (2010). The potential of virtual reality and gaming to assist successful aging with disability. Phys Med Rehabil Clin N Am.

[26] Mao Y, Chen P, Li L, Huang D (2014). Virtual reality training improves balance function. Neural Regen Res.

[27] Yang WC, Wang HK, Wu RM, Lo CS, Lin KH (2016). Home-based virtual reality balance training and conventional balance training in Parkinson’s disease: A randomized controlled trial. J Formosan Med Assoc.

[28] Yesilyaprak SS, Yildirim MS, Tomruk M, Ertekin O, Algun ZC (2016). Comparison of the effects of virtual reality-based balance exercises and conventional exercises on balance and fall risk in older adults living in nursing homes in Turkey. Physiother Theory Practice.

[29] Mousavi Hondori H, Khademi M (2014). A review on technical and clinical impact of Microsoft Kinect on physical therapy and rehabilitation. J Med Eng.

[30] Collado-Mateo D, Dominguez-Muñoz FJ, Adsuar JC, Merellano-Navarro E, Gusi E (2017). Exergames for women with fibromyalgia: a randomised controlled trial to evaluate the effects on mobility skills, balance and fear of falling. Peer J.

[31] McConville KMV, Virk S (2012). Evaluation of an electronic video game for improvement of balance. Virtual Reality.

[32] Shih MC, Wang RY, Cheng SJ, Yang YR (2016). Effects of a balance-based exergaming intervention using the Kinect sensor on posture stability in individuals with Parkinson’s disease: a single-blinded randomized controlled trial. J NeuroEng Rehab.

[33] Sisto SA, Forrest GF, Glendinning D (2002). Virtual reality applications for motor rehabilitation after stroke. Top Stroke Rehabil.

[34] Adamovich SV, Fluet GG, Tunik E, Merians AS (2009). Sensorimotor training in virtual reality: a review. NeuroRehabilitation.

[35] Bohil CJ, Alicea B, Biocca FA (2011). Virtual reality in neuroscience research and therapy. Nat Rev Neurosci.

[36] Choi JH, Kim BR, Han EY, Kim SM (2015). The effect of dual-task training on balance and cognition in patients with subacute post-stroke. Ann Rehabil Med.

[37] Newell KM, Liu YT, Mayer-Kress G (2001). Time scales in motor learning and development. Psychol Rev.

[38] Roessingh J, Hilburn B. The power law of practice in adaptive training applications. In: The annual meeting of the human factors society, UK; 2000. pp. 1–19.

[39] Heathcote A, Brown S, Mewhort DJK (2000). The power law repealed: the case for an exponential law of practice. Psychono Bull Rev.

[40] Palmieri RM, Ingersoll CD, Stone MB, Krause BA (2002). Center-of-pressure parameters used in the assessment of postural control. J Sport Rehab.

[41] Paillard T, Noe F (2015). Techniques and methods for testing the postural function in healthy and pathological subjects. BioMed Res Int.

[42] Lemay JF, Gagnon DH, Nadeau S, Grangeon M, Gauthier C, Duclos C (2014). Center-of-pressure total trajectory length is a complementary measure to maximum excursion to better differentiate multidirectional standing limits of stability between individuals with incomplete spinal cord injury and able-bodied individuals. J NeuroEng Rehab.

[43] Prieto TE, Myklebust JB, Hoffmann RG, Lovett EG, Myklebust BM (1996). Measures of postural steadiness: differences between healthy young and elderly adults. IEEE Trans Biomed Eng.

[44] Brog F. The confidence 95 ellipse. 2002. https://www1.udel.edu/biology/rosewc/kaap686/reserve/cop/center of position conf95.pdf. Accessed 4 Aug 2006.

[45] Tiedemann A, Sherrington C, Lord SR (2013). The role of exercise for fall prevention in older age. Motriz-Revista De Educacao Fisica.

[46] Silsupadol P, Shumway-Cook A, Lugade V, van Donkelaar P, Chou LS, Mayr U, Woollacott MH (2009). Effects of single-task versus dual-task training on balance performance in older adults: a double-blind, randomized controlled trial. Arch Phys Med Rehab.

[47] Hall CD, Heusel-Gillig L (2010). Balance rehabilitation and dual-task ability in older adults. J Clin Gerontol Geriatr.

[48] Wollesen B, Voelcker-Rehage C (2014). Training effects on motor–cognitive dual-task performance in older adults. Eur Rev Aging Phys Act.

[49] Pichierri, G., Wolf, P., Murer, K., de Bruin, E.D.: Cognitive and cognitive-motor interventions affecting physical functioning: A systematic review. BMC Geriatrics 11, 29 (2011). :10.1186/1471-2318-11-29 10.1186/1471-2318-11-29 PMC3147016 21651800

[50] Kubicki A, Petrement G, Bonnetblanc F, Ballay Y, Mourey F (2012). Practice-related improvements in postural control during rapid arm movement in older adults: a preliminary study. J GerontoL.

[51] Horak FB (2006). Postural orientation and equilibrium: what do we need to know about neural control of balance to prevent falls?. Age Ageing.

[52] Halvarsson A, Dohrn IM, Stahle A (2015). Taking balance training for older adults one step further: the rationale for and a description of a proven balance training programme. Clin Rehab.

[53] Barry G, van Schaik P, MacSween A, Dixon J, Martin D (2016). Exergaming (XBOX Kinect™) versus traditional gym-based exercise for postural control, flow and technology acceptance in healthy adults: a randomised controlled trial. BMC Sports Sci Med Rehab.

[54] Kalron A, Fonkatz I, Frid L, Baransi H, Achiron A (2016). The effect of balance training on postural control in people with multiple sclerosis using the CAREN virtual reality system: a pilot randomized controlled trial. J NeuroEng Rehab.

[55] Alotaibi AZ, Alghadir A, Iqbal ZA, Anwer S (2016). Effect of absence of vision on posture. J Phys Ther Sci.

